# COVID-19 lockdown and its latency in Northern Italy: seismic evidence and socio-economic interpretation

**DOI:** 10.1038/s41598-020-73102-3

**Published:** 2020-10-05

**Authors:** Davide Piccinini, Carlo Giunchi, Marco Olivieri, Federico Frattini, Matteo Di Giovanni, Giorgio Prodi, Claudio Chiarabba

**Affiliations:** 1 Istituto Nazionale di Geofisica e Vulcanologia, Sezione Di Pisa, Via Cesare Battisti 53, 56125 Pisa, Italy; 2 Istituto Nazionale di Geofisica e Vulcanologia, Sezione Di Bologna, Via Donato Creti 12, 40128 Bologna, Italy; 3 Department of Economics and Management, University of Ferrara, Via Voltapaletto 11, 44121 Ferrara, Italy; 4 Istituto Nazionale di Geofisica e Vulcanologia, Osservatorio Nazionale Terremoti, Via di Vigna Murata 605, 00143 Rome, Italy

**Keywords:** Seismology, Viral infection

## Abstract

The Italian Government has decreed a series of progressive restrictions to delay the COVID-19 pandemic diffusion in Italy since March 10, 2020, including limitation in individual mobility and the closure of social, cultural, economic and industrial activities. Here we show the lockdown effect in Northern Italy, the COVID-19 most affected area, as revealed by noise variation at seismic stations. The reaction to lockdown was slow and not homogeneous with spots of negligible noise reduction, especially in the first week. A fresh interpretation of seismic noise variations in terms of socio-economic indicators sheds new light on the lockdown efficacy pointing to the causes of such delay: the noise reduction is significant where non strategic activities prevails, while it is small or negligible where dense population and strategic activities are present. These results are crucial for the a posteriori interpretation of the pandemic diffusion and the efficacy of differently targeted political actions.

## Introduction

The extreme effort of the Italian Government to prevent or delay the diffusion of the COVID-19 resulted in the lockdown of social, cultural, and part of economic and industrial activities over the entire country starting from March 10, 2020 (https://www.gazzettaufficiale.it/eli/gu/2020/04/11/97/sg/pdf hereinafter DPCM-1) imposing social distancing to the entire population. A second decree, on March 22, extended the closure to all the non-strategic economic activities while few remained fully operative (https://www.gazzettaufficiale.it/eli/gu/2020/03/22/76/sg/pdf hereinafter DPCM-2). Besides social and economic effects and extensive daily life disruption (any unnecessary individual circulation was prohibited), restrictions also determined a decrease in the seismic ambient noise due to the integrated effects of natural (ocean waves and wind) and anthropogenic sources^1–3^.

As seismic monitoring networks operate continuously, seismic data can be easily used to track changes in the ambient noise. This approach is effective in providing insights on the variation of natural and anthropogenic noise sources^4^, in seasonality of waves and winds^5^, and on the effect of disruptive events like the Super Typhoon Ioke^6^. The observation of the anthropogenic noise at high frequency (F > 1 Hz) dates back to the 60′s^7^ and, especially in regions with high anthropic activity and poor rock characterization like a large portion of our study area (i.e. the alluvial basin of the Po Plain), ambient noise is high and masks low magnitude seismicity^8^. Anthropogenic sources are mainly associated with road and rail traffic, cultural and industrial activity causing a characteristic pattern of two superimposed fluctuations with daily and weekly periodicity [^3^, and references therein]. This effect is modulated at each site according to the ratio between anthropogenic and natural noise and to the source-receiver distance and confirmed by the observed correlation between economic conditions and noise level at global scale^9^.

The general trend of ambient noise reduction after the lockdown has been first described for Shillong (India)^10^, for Northern Italy by Poli et al.^11^ and, at global scale, by Lecocq et al.^12^. In this work, we focus with higher detail on the regional-scale transition across the March 10 lockdown (DPCM-1), with a combined analysis of time-varying seismic ambient noise and static socio-economic dimensions, such as population density and composition of economic activities (strategic and non-strategic industries). Moreover, with respect to^10–12^, we tackle the causes for the observed spatial and temporal fluctuations in noise reduction with the goal of understanding how they originated.

**Figure 1:**
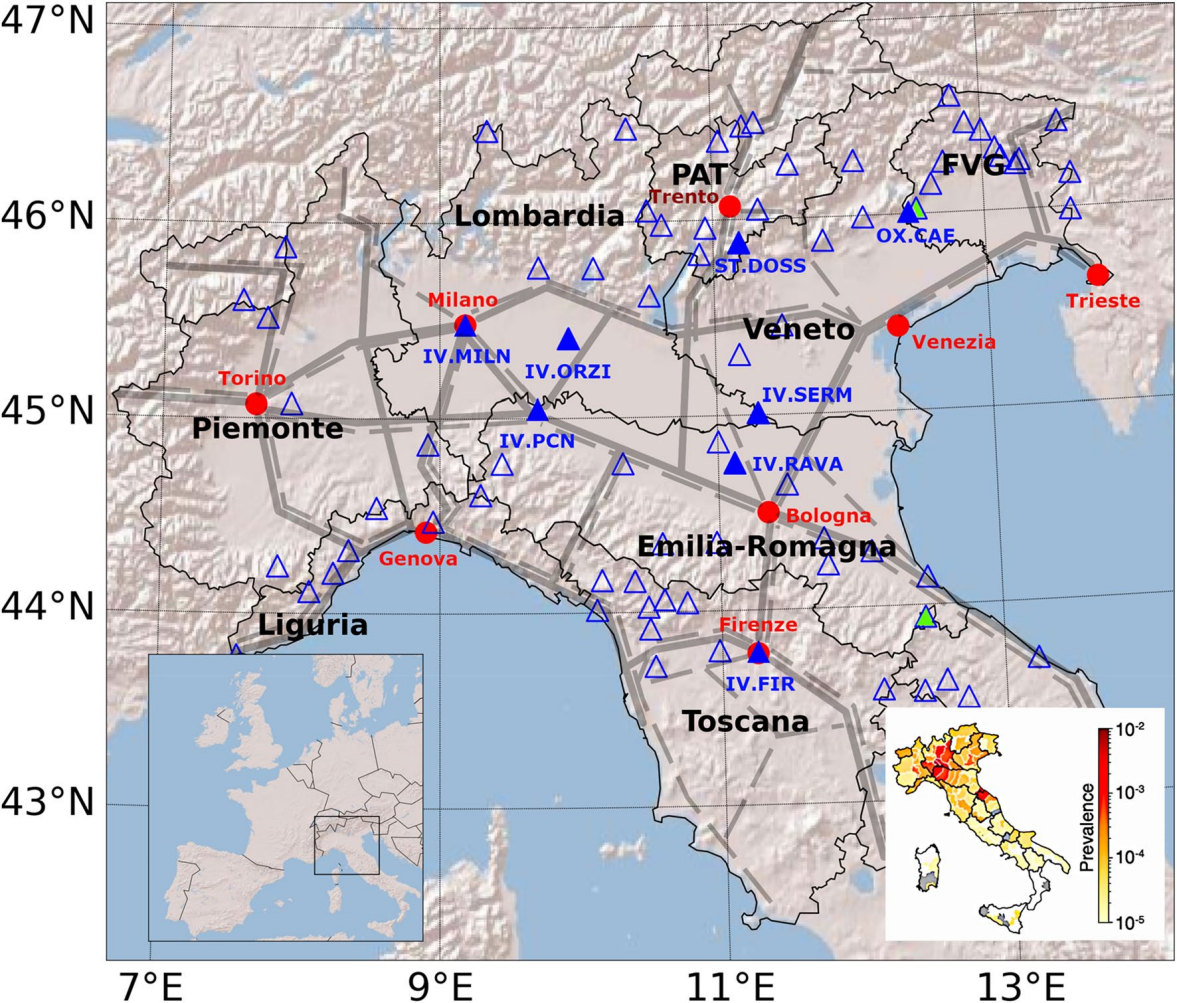
Map of the Italian regions targeted by this work. Thin black lines are regional borders. The bottom left panel shows where these regions are located in Western Europe. Blue-filled triangles mark the location of the stations discussed in the “Results” section. Empty triangles mark the location of all the seismic stations considered in this work. Green filled triangles mark those sites excluded from the socio-economic analysis. Red-filled circles mark the location of regional capital cities. PAT and FVG stand for Provincia Autonoma di Trento and Friuli-Venezia Giulia, respectively. Gray lines marks highways (solid) and railways (dashed). Figure created using Matplotlib Basemap Toolkit^41^. Bottom right frame: COVID-19 spatial spread as confirmed case/population in Italy for March 10, 2020. Figure modified after Gatto et al.(^18^: figure 1, used under CC BY 4.0 https://creativecommons.org/licenses/by/4.0/).

**Figure 2:**
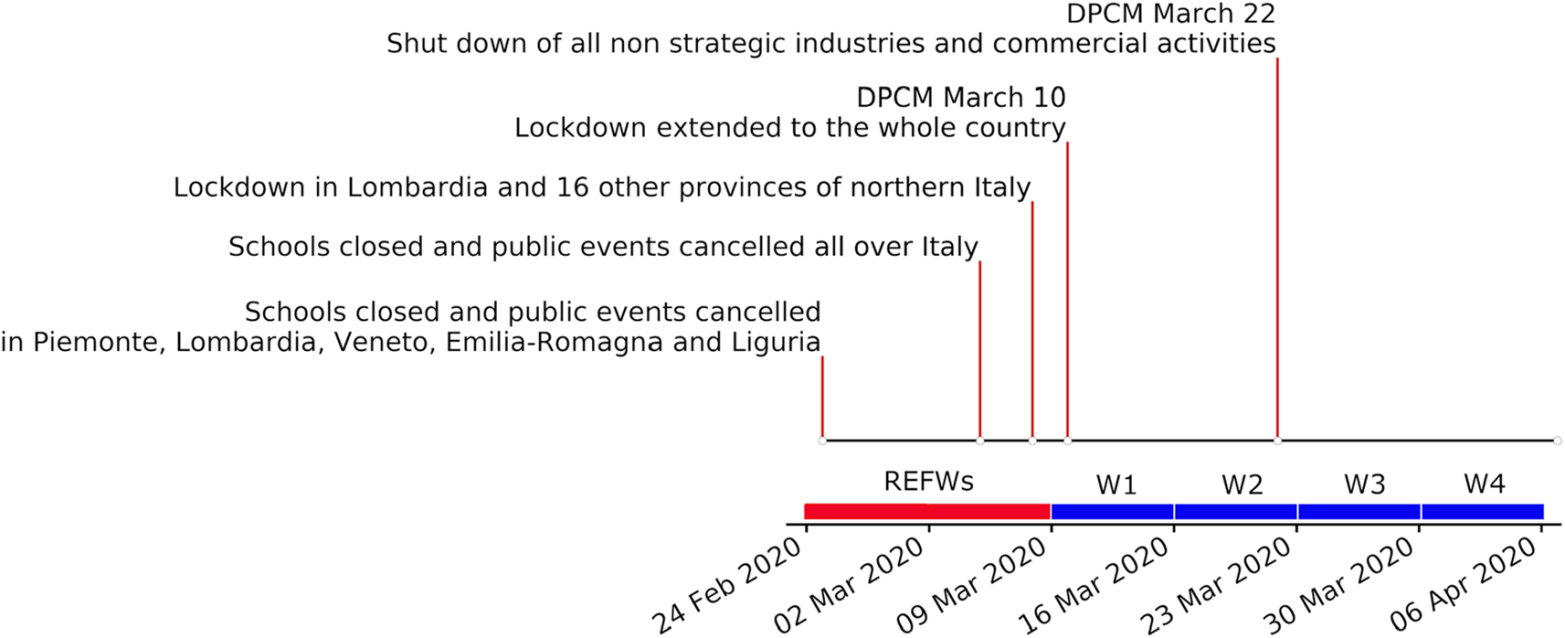
Timeline of the governmental actions and of the impact of Covid-19 in Italy. On the time axis we highlighted the weeks considered in this work: two before lockdown (red) and four after (blue).

The seismic dataset for Northern Italy is based on the recordings of 78 seismic stations (Fig. 1) during 6 weeks across March 10 (timeline in Fig. 2 and details in the “Material and methods” section). The two weeks before the lockdown (Feb 24—March 7, 2020) are used as a baseline (REFWs), while the four subsequent weeks (March 8-April 5, 2020) will be referred as W_j_, with j = 1,4. We will focus on the frequency band for 5 to 20 Hz and retrieve, for each site, a time series of the displacement noise amplitude. This choice, discussed in details in “Methods” section, stems from the need of representing the ambient noise variations with a unit measure readily understandable by a broad scientific community.

## Results

We first show noise amplitude time series, expressed as ground displacement, for some representative sites to focus on how and when noise level changed over time. When available, these are also compared with traffic data from local municipalities or other sources and with NO_2_ pollutant from local environmental protection agencies. We remark here that the quantitative correlation between pollutants, vehicular traffic and noise would require a dedicated work also accounting for the changes of meteorological conditions over time. The availability of further data characterizing the “recovery” following the lockdown conclusion would make such a study even more interesting.

**Figure 3:**
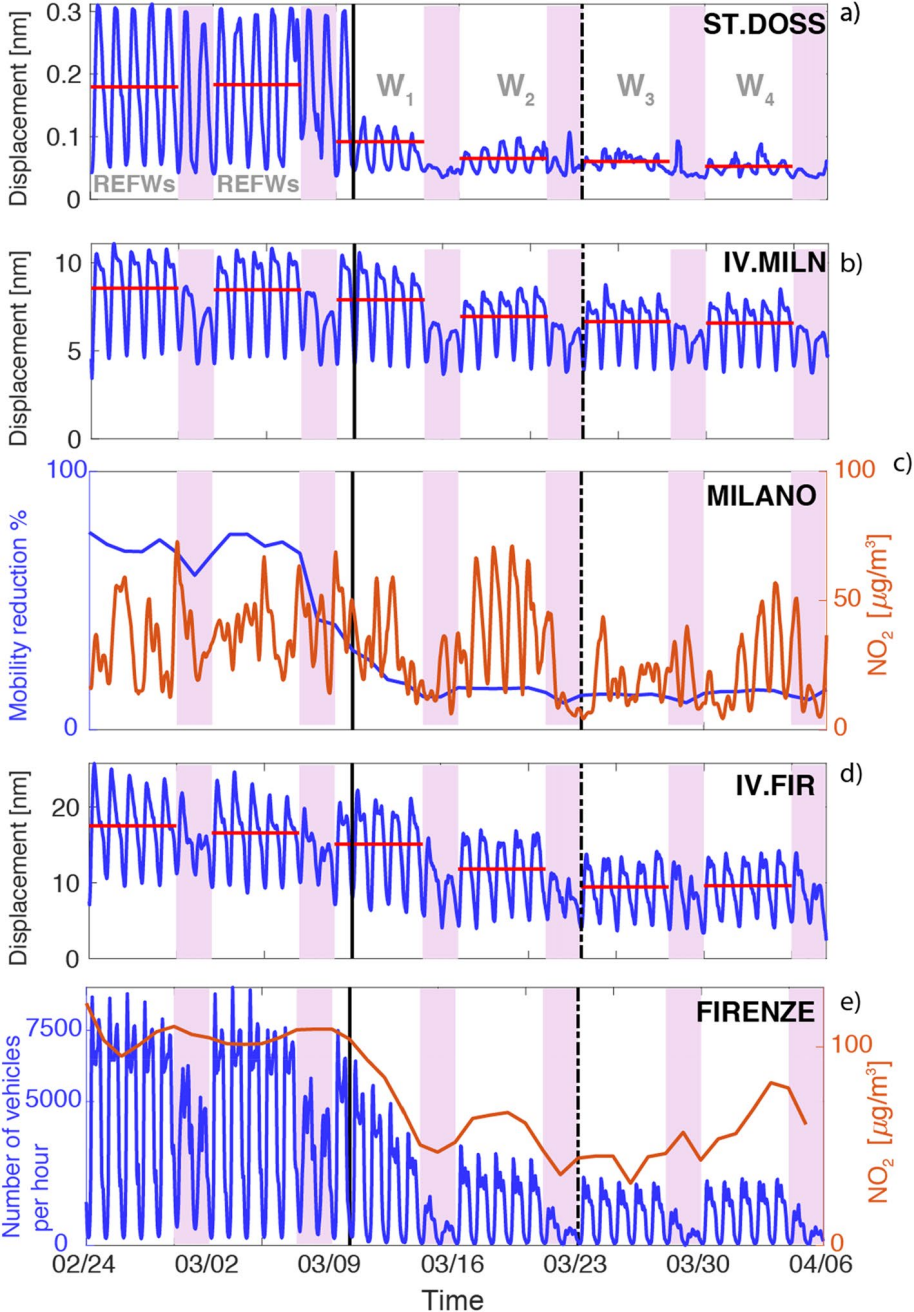
(**a**, **b**, **d)** Time series of the noise amplitude (in nm) in the frequency band between 5 and 20 Hz as obtained from the PPSD analysis. Red horizontal lines represents the average noise level for each working week. Continuous black vertical line mark the lockdown date (DPCM-1) while the following dashed one refers to the closure of all commercial activities (DPCM-2). Light purple vertical bands highlight the weekend. In (**a**) in light gray we indicate the reference time window represented by the 2 weeks preceding the lockdown (REFWs) and the following four weeks (W_1_–W_4_). In (**c**) we show using blue and orange lines, mobility reduction and NO_2_ level respectively as recorded at Milano. In (**e**) the number of vehicles per hour (blue line) and NO_2_ level (orange line) for Firenze are shown. Further details could be found in section “Results”.

The noise recording of the seismic station ST.DOSS is the first example considered (Fig. 3a). This station is sited in the Alpe Cimbra (Folgaria, see Fig. 1), one of the most popular ski districts in Italy. As a consequence of the lockdown, the ski slopes, lifts, cableways, mountain lodges and restaurants have been closed and, as clearly recognizable from Fig. 3a, the noise level drop is large, sudden and persistent over the weeks, showing very small daily and weekly fluctuations. The computed percent noise variation (PNV, Eq. 2) is equal to − 50% during W_1_, to − 64% during W_2_, to − 66% for the following W_3_ and finally it reaches − 71% during W_4_. This site represents the simplest and more effective case of quieting, since here the source of anthropogenic noise is just one ski resort and the on–off effect is perfectly represented since the few activities were closed simultaneously. Similar features are visible in other remote sites in the proximity of alpine ski resorts.

The second and opposite case is IV.MILN (Fig. 3b), sited in the centre of Milano, the most densely populated city in Northern Italy (~ 7400 people/km^2^), with large commuting rates (Central station serves on average 320,000 people per day) and with the largest public transport system in Italy (4 underground lines, 80 transit bus lines and 18 tramways). Although data on people mobility shows a sharp decrease since W_1_ (data source https://www.apple.com/covid19/mobility), the noise variation is moderate over W_1_-W_4_ and PNV reaches a small − 20% in W_4_. Nonetheless, a strong reduction in the day/night fluctuation is visible (Fig. 3b) and the lockdown also affects the peak-to-peak amplitude of the Saturday noise level that, after DPCM-1 (black line in Fig. 3), becomes comparable to the noise recorded during Sunday, when large part of tertiary activities are closed. The persistency of high noise levels over the week can be attributed to public (and private) transport traffic, consistent with the high level of NO_2_^13^, commonly considered a proxy for the rate of circulating petrol vehicles (Fig. 3c).

Topologically similar is the station IV.FIR (Fig. 3d), sited in Firenze city centre (population ~ 400,000, density 3700 people/km^2^), one of the most visited tourist attraction in Italy (more than 15.8 million visitors in 2019, data source Città Metropolitana di Firenze, Statistica del Turismo). The average noise progressively decreases after the lockdown with PNV = − 11% during W_1_ and almost reaching − 50% during W_3_ and W_4_. This reduction is well explained by the sharp decrease of the urban traffic in terms of number of vehicles per hour (data source https://www.comune.fi.it/). The reduction of the average noise level is associated to a decrease of the night minimum, probably due to the closure of the nightlife activities in the city centre. In this case, traffic reduction reflects on the NO_2_ concentration average measured (data source https://www.arpat.toscana.it) (Fig. 3e). We also note that PNV is smaller than the daily traffic decrease (~ − 75%), this suggesting the presence of other persisting anthropogenic sources. These examples represent different responses to the lockdown, highlighting sudden variations in remote places and slow and small changes in urban environments where traffic is not the exclusive source of anthropogenic noise.

**Figure 4:**
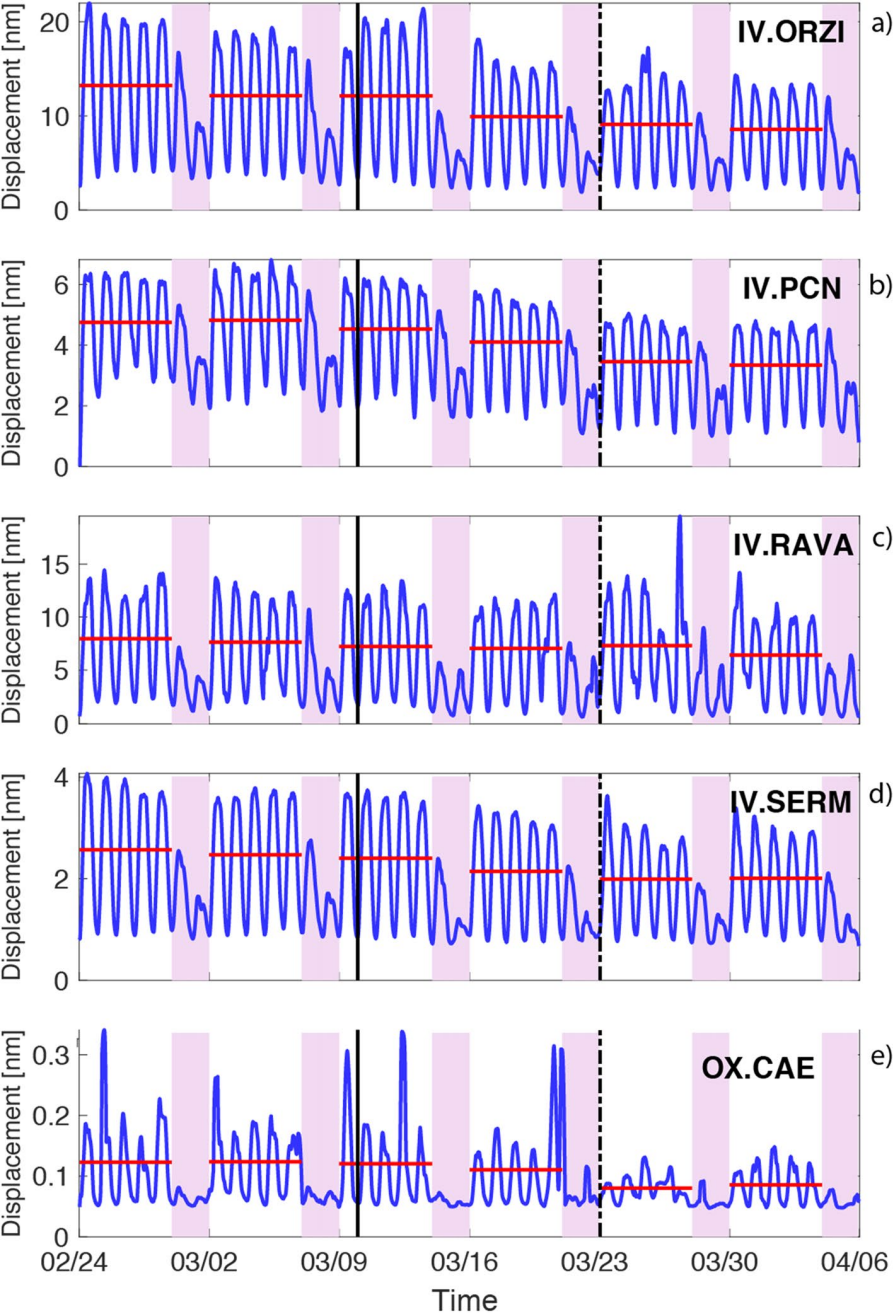
Time series of the noise amplitude (in nm) in the frequency band between 5 and 20 Hz obtained from the PPSD analysis as for (**a**–**c**) of Fig. 3.

Figure 4 shows five further seismic noise time series from sites located in different context: IV.PCN (Piacenza) is located in a medium size city (population ~ 100,000); IV.ORZI is close to Orzinuovi town (population 12,000) and sited in a densely populated neighbourhood; IV.SERM and IV.RAVA are located in a rural context, close to small villages. Despite the different characteristics, all these sites show similar behaviour in terms of PNV: an almost negligible decrease in W_1_ and W_2_ (from − 5 to 20%), that becomes visible only in W_3_ and W_4_ with PNV equal to about − 20 and − 30% respectively. In some cases, this general decrease is accompanied by a limited decrease of the noise level at night (IV.PCN, IV.ORZI, Fig. 4a,b), while this does not happen at other sites (IV.RAVA, IV.SERM, Fig. 4c,d). A further indicator of the ongoing change is the peak-to-peak amplitude in the working weeks. IV.RAVA, is, among the four discussed sites, the only one showing a small decrease and this can be consequence of the low population density at IV.RAVA (60 people/km^2^ in a 2.5 km radius) that is, on average one order of magnitude smaller than the other three sites (IV.ORZI ~ 500 people/km^2^, IV.PCN ~ 900 people/km^2^, IV.SERM ~ 300 people/km^2^). This certainly turns into a lower vehicle traffic in the area that, in addition to the lack of urban public transport, makes this contribution smaller compared to other sources like industries. Finally, at station OX.CAE (Fig. 4e), sited at the borders between Veneto and Friuli-Venezia Giulia regions, PNV is negligible during W_1_ and W_2_, while it became significant in W_3_ (− 30%). The station is close to a large industrial area where no strategic activities remained operating until DPCM-2.

**Figure 5:**
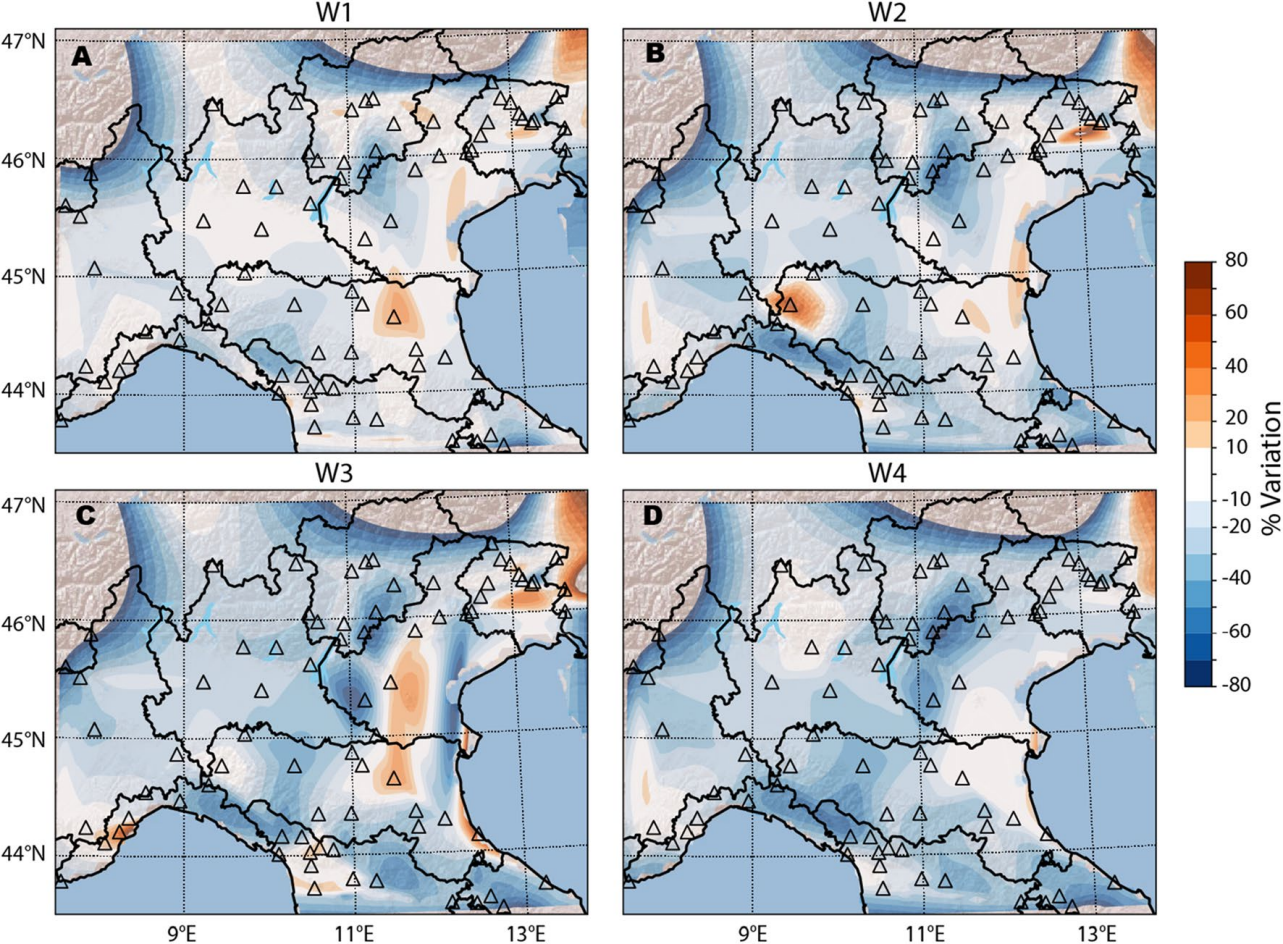
Spatial interpolation of the percent noise variation (PNV) for the case of W_1_, W_2_, W_3_ and W_4_. In each panel triangles mark the position of the 78 seismic stations, each colour coded according to its corresponding PNV and to the selected palette shown on the right side of the figure. White colour refers to a percent variation between − 10% and 10% that we consider a null change to encompass the error bars. Figure created using Matplotlib Basemap Toolkit^41^.

The general trend of ambient noise reduction after the lockdown has been presented for the Northern Italy area^11^. Here, we focus on the March 10 lockdown looking closer at spatio-temporal changes in seismic noise. To evaluate the lockdown effects at a regional scale and to visually support the following socio-economic analysis, we interpolate PNV observed at the 78 selected sites over a dense grid (see “Methods” section for details) on a weekly base. Figure 5 represents the colour coded PNV for W_1_-W_4_. White colour indicates an almost negligible variation (± 10%) with respect to REF and it also encompasses the PNV uncertainty (Eq. 3) that on average results $\le 6\%$ (see “Methods” section). During W_1_, the recognizable large white patches support the hypothesis of the absence of significant reduction probably linked to a delayed effectiveness of the lockdown. Since W_2_, PNV becomes significant in a large number of sites and this reflects in a reduced size for white area as the expected social distancing action take power. We can also recognize, for W_3_ a counterintuitive spot of increased noise in Veneto and Emilia-Romagna regions (Fig. 5c). This is the consequence of a strong wind perturbation that took place for several days^14^.

To quantify the lockdown reactions at different sites and the patterns revealed by noise spatial variations, we perform a cross-sectional analysis to understand how the effectiveness and timing of the lockdown can be related to local socio-economic structures. This dataset is preferred with respect to punctual and time dependent data like pollution or car traffic since it represents an overall picture of the different human activities and it covers the whole region with detail. At this stage the dataset is reduced to 76 sites since for two of them (NI.POLC and IV.RMS2, green triangles in Fig. 1) economic data are not available in the selected databases.

PNV does not allow to capture the typical noise levels at sites that are generated by different degrees of anthropization. For this reason, we use Noise Variation (*NV*_*i,j*_) the difference between the noise level in a post-lockdown week (*N*_*i,j*_) and the REF (Eq. 1) and we consider Population (*P*_*i*_) and Economic Activities (EA) as explanatory variable. For the latter we distinguish between Strategic and Not strategic Economic Activities introduced by DPCM-2 (*SEA*_*i*_ and *NEA*_*i*_, respectively). We observe that seismic station location could not be representative of the entire municipality as, in some cases, locations are within the city centre while others are in remote sites within the municipal territory. For this reason, we define *P*_*i*_ as the population within 2.5 km from stations, while *SEA*_*i*_ and *NEA*_*i*_ are approximated as the expected number of persons employed within the same distance. As socio-economic data (*P*_*i*_, *SEA*_*i*_, *NEA*_*i*_) do not vary over weeks, we perform four separate cross-sectional tests, one test for each week *j* after DPCM-1. Since REF varies from site to site, each regression (Eq. 7) also include *N*_*i,j*_. as explanatory variable. See “Methods” section for additional details on measures and data analysis and Supporting Information for additional details on EA grouping.

Table 1 shows the results from Ordinary Least Squares (OLS) multivariate correlation test between noise variation and socio-economic dimensions defined by Eq. (7).

**Table 1:** Each column summarizes the results from multivariate OLS regressions of noise variation *NV*_*i,j*_ against noise levels in week *j* (*N*_*i,j*_), population (*P*_*i*_), employment in strategic activities (*SEA*_*i*_) and non-strategic activities (*NEA*_*i*_), standardized explanatory variables (z-scores), p-value: * < 0.1, ** < 0.5, *** < 0.01. CI stands for confidence interval.

	*NV*_*i,*1_	*NV*_*i,*2_	*NV*_*i,*3_	*NV*_*i,*4_
$\underset{\_}{N{V}_{j}}$	− 0.17 (95% CI: − 0.23, − 0.10)***	− 0.36 (95% CI: − 0.46, − 0.27)***	− 0.38 (95% CI: − 0.65, − 0.12)***	− 0.50 (95% CI: − 0.64, − 0.35)***
*N*_*i,j*_	− 0.25 (95% CI: − 0.34, − 0.16)***	− 0.37 (95% CI: − 0.50, − 0.25)***	0.014 (95% CI: − 0.33, 0.35)	− 0.64 (95% CI: − 0.84, − 0.45)***
*P*_*i*_	0.48 (95% CI: 0.14, 0.82)***	− 0.49 (95% CI: − 0.99, 0.013)*	0.46 (95% CI: − 1.0024, 1.93)	0.84 (95% CI: 0.055, 1.63)**
*SEA*_*i*_	1.75 (95% CI: 0.73, 2.77)***	2.39 (95% CI: 0.83, 3.96)***	9.09 (95% CI: 4.73, 13.45)***	7.21 (95% CI: 4.87, 9.56)***
*NEA*_*i*_	− 2.20 (95% CI: − 3.46, − 0.95)***	− 2.09 (95% CI: − 4.02, − 0.17)**	− 9.93 (95% CI: − 15.37, − 4.50)***	− 8.14 (95% CI: − 11.059, − 5.23)***
adj-*R*^2^	0.48	0.74	0.36	0.71
*F*_(4,71)_	18.2***	54.95***	11.62***	47.25***

$\underset{\_}{NV}$ is the average noise variation in week *j*.

Although the estimated coefficient for *N*_*i,j*_ is statistically non-significant for W_3_ because of the wind storm mentioned above, the tests confirm that, on average, noise variation *NV*_*j*_ decreases over weeks, namely from − 0.17 nm (W_1_, 95% CI: − 0.23, − 0.10) to − 0.50 nm (W_4_, 95% CI: − 0.64, − 0.35). Furthermore, *SEA*_*i*_ appears to have restrained the lockdown effect, while noise reduction has been more pronounced where *NEA*_*i*_ are more agglomerated. In other words, our analysis shows that both SEA and NEA contribute to the noise lowering, but SEA contributes less than average while NEA more than average. The consistency of this evidence all over W_1_–W_4_ suggests that the limitations first introduced by DPCM-1 in W_1_ induced a different response between “core” EA (some of those identified as strategic later by DPCM-2) and other industries that chose to suspend their activity in consequence of a fast-decreasing demand. To give an example, one of the Italian retailer association^15^ claimed huge decline in goods demand for March 2020 (apparel and footwear − 67.4% automotive − 82.4%, furniture − 66.2%, and white goods and electric appliances − 54.3%.). The different contribution from the two groups of EA is then magnified after W_3_, when the separation was formally introduced by DPCM-2. When all the other variables are held constant, the effect of one standard-deviation increase of *SEA*_*i*_ (~ 81,000) on *NV*_*i,j*_ grows indeed from 1.75 nm in W_1_ (95% CI: 0.73, 2.77) to 7.21 nm in W4 (95% CI: 4.87, 9.56). On the opposite, the effect of one standard-deviation increase of *NEA*_*i*_ (~ 42,000) is − 2.20 nm in W_1_ (95% CI: − 3.46, − 0.95) and − 8.14 nm in W4 (95% CI: − 11.059, − 5.23). The only uneven result is the effect estimated for one standard-deviation increase of *P*_*i*_ (~ 24,000), which is positive in W_1_ (0.48, 95% CI: 0.14, 0.82) and W_4_ (0.84, 95% CI: 0.055, 1.63), null in W3 (0.014, 95% CI: − 0.33, 0.35), and negative in W_2_ (− 0.37, 95% CI: − 0.50, − 0.25). The adj-*R*^2^ obtained from the ratio between the variance captured by the explanatory variables and the total variance of *NV*_*i,j*_ shows that the explanatory power of the tests varies over weeks, and the highest in W_2_ (0.74) and W_4_ (0.71). Nonetheless, statistically significant *F*-statistics confirm that the set of explanatory variables is meaningful for all the four tests.

## Discussion

Spatial patterns of noise variation reflect the heterogeneous landscape captured by the sparse stations, where many overlapping sources concur to the overall signal and qualitatively well compare with^11^. Among the others, industrial machinery, buildings and urban traffic dominate the seismic noise spectra at frequencies larger than 1 Hz, with signal amplitudes that scale with source size and distance. In our study, we use the spectral band from 5 to 20 Hz to preserve the full set of noise sources, even though it is not possible to discriminate between different active sources in each case. We notice that sites IV.MILN and IV.FIR have been also analyzed in detail in^11^. Despite the different choice for frequency band and units, visual comparison confirms the consistency of the results.

Previous studies revealed that the noise generated by human infrastructures can be clearly observed at distances larger than 10 km, nevertheless subsurface properties may significantly impact on the distance over which noise propagates^16,17^. Since the average interstation distance is around 32 km (figure S2), values at specific sites are indeed representative of the average noise level in a broader area around the observation point, especially where the density of stations is higher and the characteristics of noise are similar.

Despite the limitations discussed so far, clear patterns of noise variation following the lockdown imposed to prevent the COVID-19 pandemic diffusion, emerge and they appear to be consistent, on average, with a reliable societal response to the emergency. A general noise reduction is apparent at most sites, but a consistent drop (PNV < − 20%) is achieved only at W_3_ (Fig. 5c). This evidence is locally very heterogeneous, varying from a sharp decrease to a progressive lowering, a small reduction that contrasts the few cases of local increase of noise level. Sharp decrease is common for remote sites (type 0 in Table S1) as those ski resorts where the lockdown brought an immediate stop of any activity (e.g. ST.DOSS). Progressive decrease is visible in large city centres such as Milano or Firenze in which the PNV reaches − 22% and − 50% during W_3_ and W_4_ respectively. Furthermore, we could observe at spot of homogeneous noise variation in the area in which COVID-19 diffusion^18^ was more pronounced (e.g. IV.RAVA, IV.SERM, IV.PCN, IV.ORZI and IV.MILN). For these sites indeed we record a slow and small decrease of the seismic noise with maximum reductions in the range between − 18% (IV.RAVA) and − 30% (IV.PCN).

The comparison between mobility and seismic data suggests a complex relationship between them. This because many vehicles, especially public transport, continued to circulate. Furthermore, this discrepancy stems from the restriction to group and family mobility, while individuals were allowed to move alone for a restricted number of needs.

Sites like IV.FIR (centre of Firenze) show that noise drop is large and persistent (Fig. 3d,e) where the traffic is the dominant source. Conversely, in sites where the drop is low, the dominant noise component could be other than private traffic and people circulation. The persistent noise could be explained by the unrest of the economic activities, as evidenced by the cross-correlation study. The correlation between *NV*_*i,j*_ and EA is coherent with the restrictions introduced by the Italian Government: the sign changes are consistent all over W_1_–W_4_ and the effect size is larger after W_3_. This suggests that some companies in non-strategic economic sectors might have decided to go offline before March 22^15^, and that the identification of *SEA*_*i*_ is, at least in part, consistent with this evolution. On the other hand, the correlation between *NV*_*i,j*_ and *P*_*i*_ is less clear and opens to some hypotheses about how people have complied with the lockdown restrictions. People may have taken some days, indeed, to fully adapt to the pandemic emergency and many services tightly proportioned to the population density, such as public transport, actually took some days to adjust their offer. Moreover, the demand for other services, such as food and good delivery might have further increased in W_4_ as evidenced by e-sales in which consumer goods registered a + 162% in W_3_ with respect to the same week in 2019^19^. We cannot exclude, however, that people might have also loosened their more general compliance with restrictions after some weeks of quarantine.

Our results highlight the compliance of societal behaviour following DPCM-1 and -2 in Northern Italy, as emerges from this innovative approach that integrates seismic data with socio-economic analysis. We document that the quieting took place slowly and not homogeneously, with areas where economic activities prevails dropping slower than others. Ambient noise variation is anyway not observed before March 10 in all sites.

Although a direct relation between noise variation and social distancing is not immediate, our results imply a delay in the change of societal attitude in the area mostly affected by COVID-19. The significant drop achieved lately at the end of March could have promoted decisive and beneficial effects in the infection rate restraint starting from mid-April.

Results from the combined analysis of seismic noise data and socio-economic markers are not only crucial supporting material for a posteriori interpretation of the COVID-19 pandemic diffusion especially for highly populated and industrial areas worldwide, but also provide further fundamentals to foresee the effectiveness of political actions and proactive measures against possible future spread of new pandemics.

## Methods

The stations currently contributing to the seismic surveillance of Italy are more than 500, belonging to different networks and only partly deployed and maintained by INGV itself^20–23^. This implies a large heterogeneity in sensors, digitizers, data transmission and siting quality. To maximize the station coverage for this study, we use the whole set of existing stations for the northern Italy (Latitude greater than 43.5, rectangle in Fig. 1) for which continuous data for the entire period of this study (February 24–April 5, 2020) are available. To better encompass all the possible sources of cultural and anthropogenic noise, we analyse the frequency band between 5 and 20 Hz, which mainly characterizes the noise produced by public and private traffic and industrial activities^24–27^. Previous studies identified road traffic noise in the frequency band 3–25 Hz while a large variety of industrial activities affect the 2–10 Hz frequency band^28^. Since other sources of non-anthropic noise, such as wind, can be observed over a broad frequency band with large differences between sites, we decided to cut out the lower frequencies (F < 5 Hz) to limit the effect of non-anthropic fluctuation on data^29^.

From the vertical component of the seismograms recorded at each site, we computed the probabilistic power spectral density (PPSD) using the method of McNamara et al.^30^. The PPSD was calculated on 1 h-long segments with a 50% overlap after the instrument correction removal. Following Lecocq et al.^12^ we convert PPSD acceleration amplitudes to displacement dividing each PPSD by (2πf)^2^ where *f* is the frequency and then, using the Parseval's identity, we obtain the displacement RMS value in the 5–20 Hz frequency range for each hour long segment. Following this approach we get a continuous time-series that covers the time span from February 24 to April 4. After removing those few stations for which the post processing analysis evidenced anomalies (e.g. Figure S1), the final dataset consists of 78 time series from stations that have good quality raw data and provide consistent time series. Spatial distribution analysis shows an interstation distance (i.e., the average distance between the two closest stations) that varies between 8 and 98 km with an average of 32 km and standard deviation equal to 14 km (Figure S2).

We estimate the mean noise level during each working week averaging the obtained time series over the Monday to Friday period for each week (Table S1). We then define a reference level REF as the average of the two weeks preceding the lockdown (REFWs) and finally we compute, for each week W_j_, the noise variation (NV) and the percent noise variation (PNV) as follow:

1 $$N{V}_{i,j}={N}_{i,j}-RE{F}_{i}$$

2 $$PN{V}_{i,j}= \frac{{N}_{i,j}-RE{F}_{i}}{RE{F}_{i}}$$

where N ≤ is the noise level at site *i* in W_*j,*_ and REF_i_ is the reference level at site *i*.

For the spatial interpolation, we choose the Delaunay triangulation^31^ because, as evidenced by Olivieri and Spada^32^ this produces the simplest network by connecting the input dataset (latitude, longitude, PNV) over the selected domain^33^. This choice reflects the idea of keeping the approach as simple as possible. We use a modified version of the original method^31^, implemented into the package *sphinterpolate* part of the Generic Mapping Tools^34^. PNV values are interpolated for each week W_1_-W_4_ by enabling tension in order to preserve local monotonicity and convexity while smoothing is performed by means of global gradient.

To provide a figure for the error associated to each PNV we define $\Delta {N}_{i,j}$ as the standard deviation of the mean for the corresponding site and week. By applying error propagation rules^35^ we obtain:

3 $$\Delta PN{V}_{i,j}= \sqrt{\frac{\Delta {{N}_{i,j}}^{2}+ \Delta RE{{F}_{i}}^{2}}{({N}_{i,j} -RE{F}_{i}{)}^{2}} +\frac{\Delta RE{{F}_{i}}^{2}}{RE{{F}_{i}}^{2}}}*PN{V}_{i,j}$$

where

4 $$\Delta RE{F}_{j} = \frac{ \sqrt{(\Delta {N}_{REFW1}{)}^{2} +(\Delta {N}_{REFW2}{)}^{2}}}{2}$$

Socio-economic analysis is based on data on population and employment at a municipality scale retrieved from Istat^36^.

Demographic and economic information are updated in year 2019 and 2017, respectively. Nonetheless, we observed that the location of the stations could not be representative of the entire municipality. Some of the stations are in the city centre, while others are located in far more remote sites. In order to characterize the degree of anthropization at each site, we used data from WorldPop^37^ and computed the total number of people living within a radius of 2.5 km from the stations (*P*_*i*_). Under the reasonable hypothesis that economic activities are distributed similarly to the population, we used the ratio between the population density at 2.5 km (*PD*^25^_*i*_) and the overall population density within the municipality (*PD*_*i*_) as a scaling factor for the number of people employed (*EMP*_i_^*^) to obtain a proxy for *SEA*_*i*_ and *NEA*_*i*_ in the surroundings of the stations as follows:

5 $$SE{A}_{i}=\frac{P{{D}^{25}}_{i}}{P{D}_{i}}EM{{P}^{SEA}}_{i}$$

6 $$NE{A}_{i}=\frac{P{{D}^{25}}_{i}}{P{D}_{i}}EM{{P}^{NEA}}_{i}$$

We set four independent OLS multivariate correlation tests, one for each W_1_–W_4_, to fit a linear combination of the selected variables that best represent the distribution of *NV*_*i,j*_^38^. More formally, the test consists in four cross-sectional regressions ($j=\mathrm{1,4})$ of the following linear equation:

7 $$N{R}_{i,j} = {b}_{0} + {b}_{1}{N}_{i,j} + {b}_{2}{P}_{i} + {b}_{3}SE{A}_{i} + {b}_{4}NE{A}_{i} + {e}_{i,j}$$

where *b*_*n*_ are the estimated coefficients and *e*_*i,j*_ the error term. We remark that while *NV*_*i,j*_ and *N*_*i,j*_ are week dependent, *P*_*i*_, *SEA*_*i*_ and *NEA*_*i*_ are not. All the explanatory variables (*X*) enter the regression model in z-scores (E(*X*) = 0 and sd(*X*) = 1), so that the estimated intercept *b*_0_ is the mean of *NV*_*i,j*_. The other coefficients *b*_–0_ measure the effect of a one standard-deviation increase of *X* on *NV*_*i,j*_. Regressions have been performed with package R^39^.

The distinction between SEA and NEA is based on the list of sectors, part of DPCM-2, in which the Italian Government identified a number of economic activities (Table S3) that, observing rigorous protocols, could continue operating because strategic for the country. On the opposite, all the other (non-strategic) activities were forced to shut down. There were a few exceptions, such as for those companies proving their business as crucial to some of the SEA, but these exceptions do not substantially limit the main separation of the EA into two groups. Data collection is based on the NACE Rev.2 classification at a three-digit level^40^.

## Supplementary information

Supplementary Information 1.

## References

[1] Fratti GE (1963). The nature of high frequency earth noise spectra. Geophysics.

[2] Stutzmann E, Roult G, Astiz L (2000). GEOSCOPE station noise levels. Bull. Seismol. Soc. Am..

[3] Bonnefoy-Claudet S, Cotton F, Bard P (2006). The nature of noise wavefield and its applications for site effects studies. Earth Sci. Rev..

[4] Diaz J, Schimmel M, Ruiz M, Carbonell R (2020). Seismometers within cities: a tool to connect Earth Sciences and society. Front. Earth Sci..

[5] Demuth JL, Morss RE, Lazo JK, Trumbo C (2016). The effects of past hurricane experiences on evacuation intentions through risk perception and efficacy beliefs: a mediation analysis. Weather Clim. Soc..

[6] Gerstoft P, Bromirski PD (2016). “Weather bomb” induced seismic signals. Science.

[7] Kanai K, Tanaka T (1961). On microtremors. VIII Bull. Earthq. Res. Inst..

[8] Marzorati S, Bindi D (2006). Ambient noise levels in north central Italy. Geochem. Geophys. Geosyst..

[9] Hong T-K, Lee J, Lee G, Lee J, Park S (2020). Correlation between ambient seismic noises and economic growth. Seismol. Res. Lett..

[10] Somala SN (2020). Seismic noise changes during COVID-19 pandemic: a case study of Shillong, India. Nat. Hazards.

[11] Poli P, Boaga J, Molinari I (2020). The 2020 coronavirus lockdown and seismic monitoring of anthropic activities in Northern Italy. Sci. Rep..

[12] Lecocq T (2020). Global quieting of high-frequency seismic noise due to COVID-19 pandemic lockdown measures. Science.

[13] Arpalombardia. https://www.arpalombardia.it. Accessed 11 May 2020.

[14] Allerta Meteo Emilia-Romagna. https://allertameteo.regione.emilia-romagna.it/web/guest/singola-allerta/-/asset_publisher/FZPQSb6AzKtJ/Allerta-Bollettino/id/1096287#.XqJ3sJMzYWo. Accessed 11 May 2020.

[15] Ufficio Studi Confcommercio. https://www.confcommercio.it/documents/20126/2678762/Congiuntura+Confcommercio+%28CC%29+4-2020.pdf/86ca9f76-1e00-9757-e039-85169341296c. Accessed 11 May 2020.

[16] Saccorotti G, Piccinini D, Cauchie L, Fiori I (2011). Seismic noise by wind farms: a case study from the Virgo gravitational wave observatory, Italy. Bull. Seismol. Soc. Am..

[17] Brenguier F, Boué P, Ben-Zion Y (2019). Train traffic as a powerful noise source for monitoring active faults with seismic interferometry. Geophys. Res. Lett..

[18] Gatto M, Bertuzzo E, Mari L, Miccoli S, Carraro L, Casagrandi R, Rinaldo A (2020). Spread and dynamics of the COVID-19 epidemic in Italy: effects of emergency containment measures. Proc. Natl. Acad. Sci..

[19] Nielsen, MarketTrack. https://www.nielsen.com/us/en/insights/article/2020/covid-19-tracking-the-impact-on-fmcg-and-retail/. Accessed 11 May 2020.

[20] INGV Seismological Data Centre. *Rete Sismica Nazionale (RSN)*. Istituto Nazionale di Geofisica e Vulcanologia (INGV), Italy. 10.13127/SD/X0FXNH7QFY (2006).

[21] Geological Survey-Provincia Autonoma Di Trento. *Trentino Seismic Network*. International Federation of Digital Seismograph Networks. 10.7914/SN/ST (1981).

[22] OGS (Istituto Nazionale Di Oceanografia E Di Geofisica Sperimentale). *North-East Italy Seismic Network*. International Federation of Digital Seismograph Networks. 10.7914/SN/OX (2016).

[23] University Of Genova. *Regional Seismic Network of North Western Italy*. International Federation of Digital Seismograph Networks. 10.7914/SN/GU (1967).

[24] Nakata N, Snieder R, Tsuji T, Larner K, Matsuoka T (2011). Shear-wave imaging from traffic noise using seismic interferometry by cross-coherence. Geophysics.

[25] Riahi N, Gerstoft P (2015). The seismic traffic footprint: tracking trains, aircraft, and cars seismically. Geophys. Res. Lett..

[26] Bormann, P. & Wielandt, E. *Seismic Signals and Noise*, Version June 2013 (2013).

[27] Holub K (1998). Some made man sources of the seismic noise. Acta Montana IRSM AS CR Ser. A.

[28] Groos JC, Ritter JRR (2009). Time domain classification and quantification of seismic noise in an urban environment. Geophys. J. Int..

[29] Lott FF, Ritter JRR, Al-Qaryouti M (2017). On the analysis of wind-induced noise in seismological recordings. Pure Appl. Geophys..

[30] McNamara DE, Buland RP (2004). Ambient noise levels in the continental United States. Bull. Seismol. Soc. Am..

[31] Brassel KE, Reif D (1979). A procedure to generate Thiessen polygons. Geograph. Anal..

[32] Olivieri M, Spada G (2016). Spatial sea-level reconstruction in the Baltic Sea and in the pacific Ocean from tide gauges observations. Ann. Geophys..

[33] Manni F, Guerard E, Heyer E (2004). Geographic patterns of (genetic, morphologic, linguistic) variation: how barriers can be detected by using Monmonier’s algorithm. Hum. Biol..

[34] Wessel P, Smith WHF, Scharroo R, Luis JF, Wobbe F (2013). Generic mapping tools: improved version released. EOS Trans. AGU.

[35] Taylor JR (1997). An Introduction to Error Analysis: The Study of Uncertainties in Physical Measurements.

[36] Istat (Istituto Nazionale di Statistica).*Online data warehouse*. https://www.istat.it. Accessed 23 April 2020.

[37] WorldPop (www.worldpop.org - School of Geography and Environmental Science, University of Southampton; Department of Geography and Geosciences, University of Louisville; Departement de Geographie, Universite de Namur) and Center for International Earth Science Information Network (CIESIN), Columbia University (2018). Global High Resolution Population Denominators Project - Funded by The Bill and Melinda Gates Foundation (OPP1134076). 10.5258/SOTON/WP00645.

[38] Greene WH (2018). Econometric Analysis.

[39] R Core Team. *R: A Language and Environment for Statistical Computing* (R Foundation for Statistical Computing, Vienna, 2013). https://www.R-project.org/.

[40] Eurostat. https://ec.europa.eu/eurostat/documents/3859598/5902521/KS-RA-07-015-EN.PDF. Accessed 11 May 2020.

[41] Hunter JD (2007). Matplotlib: a 2D graphics environment. Comput. Sci. Eng..

