# Supplementary material for: COVID-19 lockdown and its latency in Northern Italy: seismic evidence and socio-economic interpretation
Source: Sci Rep. 2020 Oct 5;10:16487. doi: 10.1038/s41598-020-73102-3 (PMC7536181; doi:10.1038/s41598-020-73102-3)
Supplement: Supplementary file 1 — Supplementary Information 1. [file 41598_2020_73102_MOESM1_ESM.docx]

**COVID-19 lockdown and its latency in Northern Italy: seismic evidence and socio-economic interpretation**

Davide Piccinini^a^*, Carlo Giunchi^a^, Marco Olivieri^b^, Federico Frattini^c^, Matteo Di Giovanni^a^, Giorgio Prodi^c^ & Claudio Chiarabba^d^

1. Istituto Nazionale di Geofisica e Vulcanologia, Sezione di Pisa, Via Cesare Battisti 53, 56125 Pisa, Italy
2. Istituto Nazionale di Geofisica e Vulcanologia, Sezione di Bologna, via Donato Creti 12, 40128 Bologna, Italy
3. Department of Economics and Management, University of Ferrara, via Voltapaletto 11, 44121 Ferrara, Italy
4. Istituto Nazionale di Geofisica e Vulcanologia, Osservatorio Nazionale Terremoti, via di Vigna Murata 605, 00143, Roma, Italy

^*^corresponding author is Davide Piccinini, [davide.piccinini@ingv.it](mailto:davide.piccinini@ingv.it)

**Supplementary Information**

Strong local noise transients.

In some cases we recorded unexpected strong local noise disturbance at stations located in remote sites. An explanatory example comes from station IV.BOB (Figure S1), located in a woody area of northern Apennine who exhibit a marked increase of noise during week 2, producing a large spot of increased noise well visible in Figure 9. A visual inspection of the seismograms reveal the presence of strong transient during daytime of week 2. We can exclude that recorded disturbances could be attributed to severe meteorological conditions given the absence of weather alert for this area, and we suggest that the strong signals recorded could be related to local farm works (i.e. forest management activities).


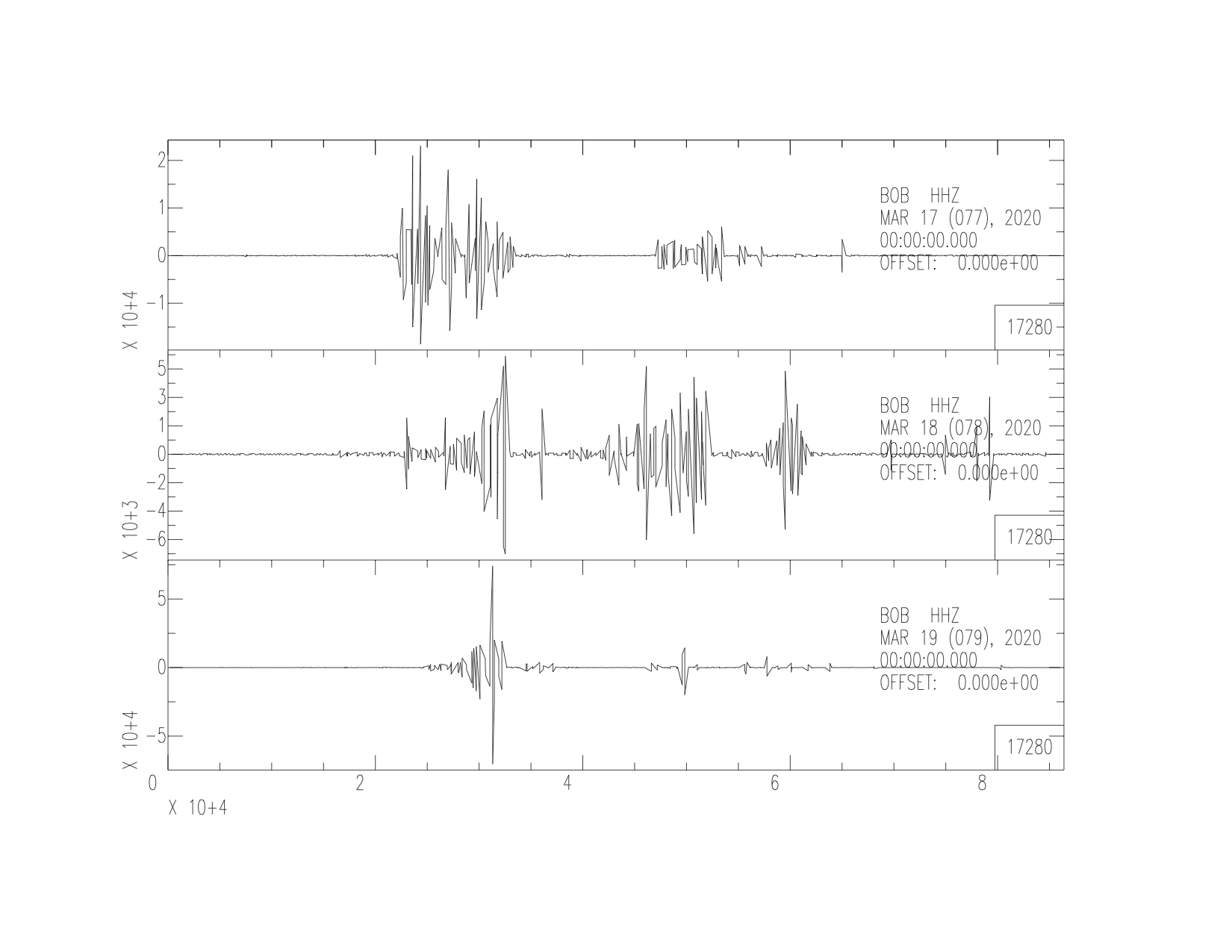


**Figure S1.** Decimated raw velocity seismograms of the vertical component (HHZ) from station IV.BOB filtered in the band 5-20 Hz for three consecutive days of W2 (each panel represent 24 hours), from March 17 to March 19. We note a pronounced local disturbance affecting the time series during daytime. Amplitude are expressed in counts.


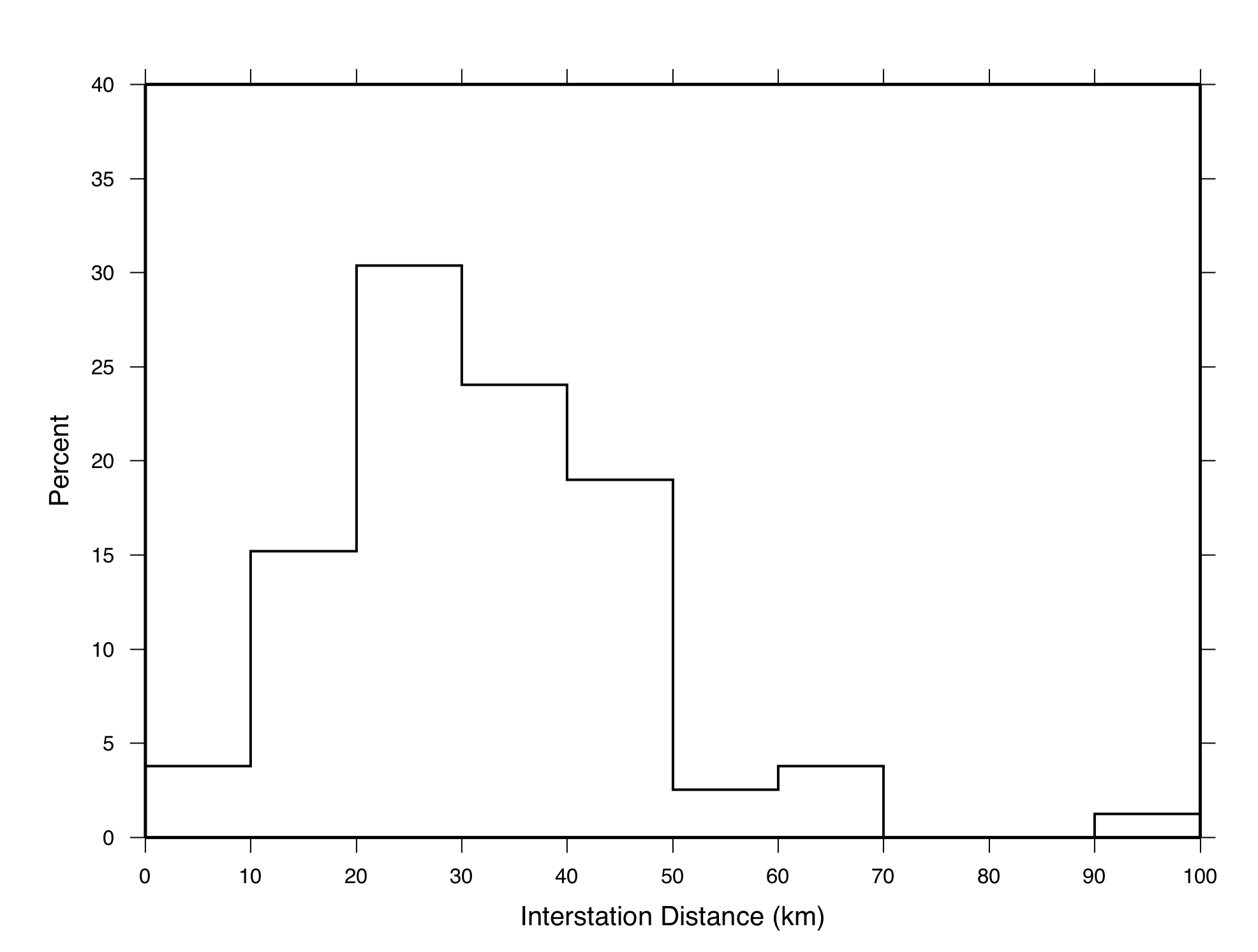


**Figure S2.** Distribution of the interstation distance for the 78 sites listed in Table S1.

**Table S1**. Noise levels has obtained from the seismic data analysis and used for computing NV and PNV. Column labelled “type” empirically defined the characteristic of the site: 0 = empty place with almost no construction in a wide range of distance, 1 = almost one small village or economic activity in the surrounding area, 2 = more than one small village or economic activity in the surrounding area, 3 = station side within a strongly urbanized area. Values for measured noise levels are in nm. R stands for radius.

| **N** | **Site** | **Lat** | **Lon** | **Elev** | **type** | **Pop**  **R< 2.5km** | **REFw1** | **REFw2** | **W_1_** | **W_2_** | **W_3_** | **W_4_** |
| --- | --- | --- | --- | --- | --- | --- | --- | --- | --- | --- | --- | --- |
| **0** | GU.BURY | 43.7825 | 7.5569 | 20 | 1 | 1143 | 0.70 | 0.98 | 0.60 | 0.47 | 0.58 | 0.38 |
| **1** | GU.CANO | 44.2075 | 8.2372 | 638 | 0 | 998 | 0.43 | 0.36 | 0.35 | 0.41 | 0.60 | 0.39 |
| **2** | GU.CARD | 44.026 | 10.4821 | 380 | 2 | 3588 | 0.44 | 0.48 | 0.37 | 0.34 | 0.57 | 0.33 |
| **3** | GU.CIRO | 45.6019 | 7.5682 | 2516 | 0 | 0 | 0.16 | 0.13 | 0.11 | 0.04 | 0.04 | 0.03 |
| **4** | GU.EQUI | 44.166 | 10.153 | 350 | 1 | 461 | 0.11 | 0.26 | 0.11 | 0.10 | 0.09 | 0.08 |
| **5** | GU.GBOS | 44.2416 | 7.8399 | 897 | 0 | 130 | 0.10 | 0.10 | 0.09 | 0.10 | 0.10 | 0.10 |
| **6** | GU.GORR | 44.6071 | 9.2926 | 609 | 1 | 98 | 0.21 | 0.46 | 0.29 | 0.24 | 0.21 | 0.17 |
| **7** | GU.GSCL | 44.35047 | 10.5881 | 674 | 1 | 547 | 0.36 | 0.33 | 0.29 | 0.24 | 0.27 | 0.25 |
| **8** | GU.MAIM | 43.91417 | 10.4915 | 200 | 1 | 695 | 0.12 | 0.13 | 0.12 | 0.12 | 0.11 | 0.11 |
| **9** | GU.MASSA | 44.0139 | 10.1153 | 6 | 3 | 24546 | 0.44 | 0.87 | 0.75 | 0.70 | 0.59 | 0.57 |
| **10** | GU.PCP | 44.54133 | 8.54517 | 770 | 0 | 183 | 0.05 | 0.05 | 0.05 | 0.04 | 0.04 | 0.04 |
| **11** | GU.POPM | 44.045 | 10.757 | 440 | 1 | 1015 | 0.59 | 1.08 | 0.69 | 0.52 | 0.63 | 0.59 |
| **12** | GU.RNCA | 44.4712 | 8.9512 | 235 | 2 | 2472 | 0.32 | 0.24 | 0.19 | 0.16 | 0.18 | 0.16 |
| **13** | GU.RORO | 44.11217 | 8.06617 | 260 | 1 | 329 | 0.21 | 0.28 | 0.23 | 0.20 | 0.29 | 0.20 |
| **15** | GU.SATI | 45.8753 | 7.8685 | 3005 | 1 | 0 | 0.24 | 0.24 | 0.08 | 0.04 | 0.04 | 0.03 |
| **16** | GU.TRAV | 45.51267 | 7.747 | 990 | 0 | 418 | 0.14 | 0.12 | 0.11 | 0.10 | 0.08 | 0.09 |
| **17** | IV.APEC | 43.55846 | 12.41991 | 488 | 2 | 1264 | 1.44 | 1.47 | 1.39 | 1.21 | 1.20 | 1.05 |
| **18** | IV.APPI | 46.47868 | 11.22813 | 1056 | 2 | 358 | 0.09 | 0.11 | 0.09 | 0.05 | 0.09 | 0.08 |
| **19** | IV.BDI | 44.0628 | 10.5956 | 830 | 1 | 195 | 0.25 | 0.25 | 0.20 | 0.17 | 0.30 | 0.18 |
| **20** | IV.BLLA | 44.14294 | 12.47033 | 1 | 3 | 16814 | 6.39 | 8.37 | 5.95 | 4.17 | 13.55 | 7.82 |
| **21** | IV.BOB | 44.76792 | 9.44782 | 910 | 0 | 36 | 0.30 | 0.24 | 0.23 | 0.45 | 0.30 | 0.20 |
| **22** | IV.BRIS | 44.22454 | 11.76657 | 260 | 2 | 2967 | 0.44 | 0.44 | 0.40 | 0.39 | 0.31 | 0.34 |
| **23** | IV.BRMO | 46.47616 | 10.37218 | 1380 | 2 | 4998 | 0.25 | 0.24 | 0.22 | 0.21 | 0.19 | 0.18 |
| **24** | IV.BRSN | 44.28418 | 12.08023 | 20 | 2 | 1153 | 9.92 | 9.07 | 8.24 | 8.86 | 7.07 | 8.74 |
| **25** | IV.CAVE | 44.8658 | 11.0031 | 18 | 2 | 1128 | 9.65 | 8.97 | 8.06 | 7.97 | 7.14 | 6.56 |
| **26** | IV.CRMI | 43.7956 | 10.97951 | 490 | 2 | 1005 | 0.46 | 0.48 | 0.38 | 0.36 | 0.52 | 0.38 |
| **27** | IV.FIR | 43.77437 | 11.25513 | 40 | 3 | 103410 | 17.38 | 16.55 | 15.09 | 11.79 | 9.43 | 9.59 |
| **28** | IV.FIU | 44.64031 | 11.49165 | 12 | 2 | 6629 | 3.02 | 3.05 | 3.99 | 3.30 | 4.15 | 2.99 |
| **29** | IV.FRON | 43.51777 | 12.72572 | 515 | 1 | 1315 | 0.76 | 0.42 | 0.21 | 0.18 | 0.17 | 0.16 |
| **30** | IV.FVI | 46.59658 | 12.7804 | 1024 | 1 | 408 | 0.59 | 0.35 | 0.25 | 0.17 | 0.22 | 0.19 |
| **31** | IV.IMOL | 44.35955 | 11.74248 | 27 | 3 | 9772 | 1.74 | 2.03 | 1.77 | 1.50 | 1.23 | 1.16 |
| **32** | IV.MABI | 46.05492 | 10.514 | 1853 | 0 | 0 | 0.11 | 0.11 | 0.08 | 0.07 | 0.08 | 0.08 |
| **33** | IV.MDI | 45.76972 | 9.716 | 954 | 2 | 1292 | 0.22 | 0.21 | 0.20 | 0.17 | 0.15 | 0.23 |
| **34** | IV.MILN | 45.4803 | 9.2321 | 125 | 3 | 177543 | 8.51 | 8.47 | 7.90 | 6.95 | 6.66 | 6.57 |
| **35** | IV.MONC | 45.0739 | 7.9271 | 480 | 1 | 1198 | 1.06 | 0.88 | 0.77 | 0.69 | 0.65 | 0.62 |
| **36** | IV.NARO | 43.61082 | 12.58058 | 272 | 1 | 300 | 0.35 | 0.41 | 0.30 | 0.29 | 0.20 | 0.21 |
| **38** | IV.OPPE | 45.3082 | 11.1724 | 20 | 2 | 4057 | 8.70 | 7.89 | 8.33 | 8.17 | 2.88 | 3.79 |
| **39** | IV.ORZI | 45.4056 | 9.9307 | 83 | 3 | 11458 | 13.16 | 12.14 | 12.11 | 9.92 | 9.09 | 8.57 |
| **40** | IV.PCN | 45.0353 | 9.7247 | 55 | 3 | 18473 | 4.79 | 4.82 | 4.53 | 4.11 | 3.45 | 3.35 |
| **41** | IV.PII | 43.72193 | 10.52495 | 66 | 2 | 5613 | 0.76 | 0.74 | 0.54 | 0.53 | 0.87 | 0.65 |
| **42** | IV.PRMA | 44.76374 | 10.31311 | 78 | 3 | 15143 | 4.37 | 4.22 | 3.25 | 2.62 | 2.38 | 2.33 |
| **43** | IV.QLNO | 44.32425 | 8.34592 | 547 | 2 | 1992 | 0.28 | 0.25 | 0.26 | 0.23 | 0.24 | 0.21 |
| **44** | IV.RAVA | 44.75587 | 11.1188 | 15 | 2 | 1091 | 7.88 | 7.63 | 7.22 | 7.03 | 7.30 | 6.39 |
| **45** | IV.RSM2 | 43.93769 | 12.44513 | 645 | 3 | 2 | 0.80 | 0.81 | 0.63 | 0.44 | 0.57 | 0.46 |
| **46** | IV.SALO | 45.6183 | 10.5243 | 600 | 2 | 8462 | 0.20 | 0.20 | 0.18 | 0.16 | 0.14 | 0.16 |
| **47** | IV.SARZ | 44.8673 | 8.9136 | 266 | 1 | 1117 | 2.79 | 2.52 | 2.13 | 1.89 | 2.13 | 1.92 |
| **48** | IV.SENI | 43.7052 | 13.2331 | 10 | 3 | 16529 | 3.12 | 2.90 | 2.65 | 1.95 | 1.98 | 1.64 |
| **49** | IV.SERM | 45.00997 | 11.29582 | 7 | 3 | 7443 | 2.55 | 2.47 | 2.40 | 2.14 | 1.99 | 2.01 |
| **50** | IV.SSP9 | 43.57387 | 12.13136 | 324 | 3 | 12616 | 0.50 | 0.49 | 0.44 | 0.38 | 0.34 | 0.35 |
| **51** | IV.STAL | 46.2601 | 12.7104 | 625 | 0 | 21 | 0.20 | 0.19 | 0.17 | 0.17 | 0.19 | 0.17 |
| **52** | IV.ZCCA | 44.35085 | 10.9765 | 700 | 1 | 2309 | 0.24 | 0.25 | 0.21 | 0.18 | 0.18 | 0.16 |
| **53** | IV.ZONE | 45.76358 | 10.11707 | 691 | 3 | 1046 | 1.10 | 1.04 | 0.93 | 0.88 | 0.73 | 0.78 |
| **54** | IV.ZOVE | 45.4536 | 11.4876 | 376 | 1 | 1637 | 0.32 | 0.34 | 0.24 | 0.21 | 0.37 | 0.26 |
| **55** | MN.TUE | 46.47223 | 9.34732 | 1924 | 0 | 5 | 0.11 | 0.11 | 0.10 | 0.09 | 0.09 | 0.10 |
| **56** | MN.VLC | 44.1594 | 10.3864 | 555 | 1 | 1335 | 0.41 | 0.78 | 0.48 | 0.40 | 0.38 | 0.36 |
| **57** | NI.POLC | 46.0266 | 12.5005 | 150 | 2 | 2898 | 0.60 | 0.55 | 0.51 | 0.48 | 0.44 | 0.43 |
| **58** | NI.VINO | 46.25383 | 13.2755 | 608 | 1 | 310 | 0.25 | 0.22 | 0.15 | 0.14 | 0.11 | 0.13 |
| **59** | OX.AGOR | 46.2829 | 12.0472 | 631 | 2 | 4542 | 0.28 | 0.30 | 0.32 | 0.30 | 0.24 | 0.23 |
| **60** | OX.BAD | 46.234 | 13.2438 | 590 | 1 | 1062 | 0.07 | 0.08 | 0.07 | 0.08 | 0.06 | 0.06 |
| **61** | OX.BOO | 46.3195 | 13.0984 | 444 | 2 | 790 | 0.28 | 0.28 | 0.28 | 0.24 | 0.30 | 0.21 |
| **62** | OX.CAE | 46.009 | 12.4379 | 870 | 1 | 34 | 0.12 | 0.12 | 0.12 | 0.11 | 0.08 | 0.09 |
| **63** | OX.CGRP | 45.8806 | 11.8047 | 1757 | 0 | 28 | 0.07 | 0.07 | 0.06 | 0.05 | 0.07 | 0.04 |
| **65** | OX.CLUD | 46.4569 | 12.8814 | 635 | 1 | 279 | 0.05 | 0.05 | 0.05 | 0.04 | 0.04 | 0.04 |
| **66** | OX.DRE | 46.1733 | 13.645 | 810 | 0 | 84 | 0.12 | 0.16 | 0.13 | 0.13 | 0.19 | 0.14 |
| **67** | OX.FUSE | 46.4142 | 13.0011 | 520 | 2 | 7091 | 0.17 | 0.17 | 0.17 | 0.14 | 0.18 | 0.13 |
| **68** | OX.MLN | 46.1495 | 12.6154 | 814 | 1 | 521 | 0.08 | 0.07 | 0.07 | 0.07 | 0.06 | 0.06 |
| **69** | OX.PRED | 46.4428 | 13.565 | 902 | 0 | 342 | 0.12 | 0.12 | 0.12 | 0.12 | 0.12 | 0.11 |
| **70** | OX.SABO | 45.9875 | 13.6336 | 621 | 1 | 160 | 0.13 | 0.23 | 0.13 | 0.12 | 0.14 | 0.14 |
| **71** | OX.VARN | 45.9922 | 12.1051 | 1265 | 0 | 8 | 0.09 | 0.07 | 0.07 | 0.06 | 0.07 | 0.06 |
| **72** | RF.GEPF | 46.275 | 13.1386 | 255 | 3 | 8050 | 0.99 | 0.96 | 0.85 | 0.78 | 0.78 | 0.66 |
| **73** | SI.BOSI | 46.4952 | 11.3185 | 242 | 3 | 62185 | 10.87 | 11.43 | 11.10 | 9.01 | 8.33 | 8.92 |
| **74** | SI.LUSI | 45.9595 | 10.9436 | 860 | 1 | 725 | 0.11 | 0.11 | 0.10 | 0.10 | 0.09 | 0.07 |
| **75** | ST.DOSS | 45.8808 | 11.1884 | 1660 | 1 | 182 | 0.18 | 0.18 | 0.09 | 0.06 | 0.06 | 0.05 |
| **76** | ST.OZOL | 46.404 | 11.0518 | 1219 | 1 | 2129 | 0.20 | 0.16 | 0.19 | 0.17 | 0.15 | 0.16 |
| **77** | ST.PANI | 46.0501 | 11.3341 | 1983 | 1 | 5 | 0.28 | 0.26 | 0.14 | 0.11 | 0.11 | 0.10 |
| **78** | ST.RONC | 45.9802 | 10.6228 | 1913 | 0 | 0 | 0.17 | 0.13 | 0.12 | 0.10 | 0.09 | 0.09 |
| **79** | ST.VARA | 45.826 | 10.8965 | 1735 | 0 | 0 | 0.23 | 0.21 | 0.18 | 0.16 | 0.17 | 0.14 |
| **80** | ST.ZIAN | 46.2764 | 11.5632 | 1154 | 1 | 2145 | 0.20 | 0.19 | 0.18 | 0.13 | 0.11 | 0.11 |

Table S2: Summary statistics

|  | obs | min | 25^th^ | 50^th^ | 75^th^ | max | mean | st.dev |
| --- | --- | --- | --- | --- | --- | --- | --- | --- |
| *NV_i,1_* | 76 | –1.880 | –0.133 | –0.050 | –0.010 | 0.960 | –0.165 | 0.378 |
| *NV_i,2_* | 76 | –5.180 | –0.258 | –0.090 | –0.028 | 0.270 | –0.362 | 0.823 |
| *NV_i,3_* | 76 | –7.540 | –0.253 | –0.070 | –0.010 | 6.170 | –0.385 | 1.450 |
| *NV_i,4_* | 76 | –7.380 | –0.298 | –0.090 | –0.038 | 0.440 | –0.497 | 1.167 |
| *N_i,1_* | 76 | 0.050 | 0.120 | 0.235 | 0.790 | 15.090 | 1.592 | 3.126 |
| *N_i,2_* | 76 | 0.040 | 0.118 | 0.210 | 0.720 | 11.790 | 1.395 | 2.708 |
| *N_i,3_* | 76 | 0.040 | 0.110 | 0.230 | 0.743 | 13.550 | 1.373 | 2.694 |
| *N_i,4_* | 76 | 0.030 | 0.108 | 0.200 | 0.653 | 9.950 | 1.260 | 2.439 |
| *P_i_* | 76 | 0.000 | 258 | 1076 | 4178 | 177543 | 7492 | 24326.3 |
| *SEA_i_* | 76 | 0.000 | 25.5 | 261.1 | 2112.1 | 696764.7 | 14198.5 | 81326.0 |
| *NEA_i_* | 76 | 0.000 | 34.7 | 227.0 | 2000.0 | 349284.3 | 8553.7 | 41707.6 |

Table S3: List of DPCM-2 classification codes (NACE Rev.2) and how these are used for defining strategic activities.

|  | NACE Rev.2 code |
| --- | --- |
| Included unchanged | 06, 09.1, 10, 11, 17, 18, 19, 20, 21, 22.1, 22.2, 26.6, 27.1, 28.3, 32.5, 33, 35, 36, 37, 38, 39, 42, 43.2, 45.2, 45.3, 45.4, 46.2, 46.3, 49, 50, 51, 52, 53, 55.1, 58, 59, 60, 61, 62, 63, 64, 65, 66, 69, 70, 71, 72, 74, 75, 80.1, 80.2, 81.2, 82.2, 85, 86, 87, 88 |
| truncated at the three-digit level | 13.94, 13.95, 13.96.20 (as 13.9)  14.12.00 (as 14.1)  16.24.20 (as 16.2)  23.19.10 (as 23.1)  28.93, 28.95.00, 28.96 (as 28.9)  32.99.1, 32.99.4 (as 32.9)  46.46, 46.49.2 (as 46.4)  46.61, 46.69.19, 46.69.91, 46.69.94 (as 46.6)  46.71 (as 46.7)  82.92, 82.99.2 (as 82.9)  95.11.00, 95.12.01, 95.12.09 (as 95.1)  95.22.01 (as 95.2) |
| Excluded because data not available | 01, 03, 05, 84, 94, 97 |
